# Genome-Based Development of Genus-Specific PCR Primers for *Pestalotiopsis*, *Neopestalotiopsis*, and *Pseudopestalotiopsis*

**DOI:** 10.3390/jof12030198

**Published:** 2026-03-10

**Authors:** Yui Harada, Shunsuke Nozawa, Yoshiki Takata, Celynne Ocampo-Padilla, Kyoko Watanabe

**Affiliations:** 1 Graduate School of Agriculture, Tamagawa University, Tamagawa-Gakuen 6-1-1, Machida 194-8610, Japan; 2 College of Agriculture, Tamagawa University, Tamagawa-Gakuen 6-1-1, Machida 194-8610, Japan; 3 Department of Crop Protection, College of Agriculture, Central Luzon State University, Science City of Muñoz 3120, Nueva Ecija, Philippines

**Keywords:** *Pestalotiopsis* sensu lato, specific primer, polymerase chain reaction

## Abstract

The genera *Pestalotiopsis*, *Neopestalotiopsis*, and *Pseudopestalotiopsis* share highly similar morphological characteristics. Although species within these genera are recognized as plant pathogens, their pathogenicity can differ even on the same host plant, highlighting the importance of accurate genus-level identification for effective disease management. However, reliable discrimination among these genera based solely on morphology or internal transcribed spacer (ITS) amplicon length is difficult. Therefore, molecular approaches based on ITS sequence data are required for practical and reliable genus-level identification. This study aimed to develop genus-specific PCR primers through comparative genome analysis using genus-specific gene regions identified from available genomic data. The performance of these primers was evaluated using 49 isolates obtained from banana, Japanese andromeda, loquat, rubber, and tea. The primer sets achieved an overall identification accuracy of 97%. One strain could not be assigned to *Pestalotiopsis*, which exhibited morphological characteristics inconsistent with the genus and was positioned outside the main *Pestalotiopsis* clade in phylogenetic analyses, supporting the taxonomic validity of the primer-based identification. These results demonstrate that the developed primers provide a reliable and practical tool for genus-level identification and taxonomic assignment of these morphologically similar fungal pathogens, including direct detection from infected plant tissues.

## 1. Introduction

The three genera *Pestalotiopsis*, *Neopestalotiopsis*, and *Pseudopestalotiopsis* share several common morphological characteristics. Their conidia are composed of five cells, including hyaline apical and basal cells and three pigmented median cells. Typically, three or more appendages arise from the apical cell, while a single appendage is present at the basal cell. These genera were historically treated as part of the genus *Pestalotiopsis*. Before the subdivision of the genus, when fungal classification was primarily based on morphology, the coloration of the three median conidial cells was used as a key criterion for grouping species [1,2].

With the development of molecular phylogenetic methods, classification based on genetic data became possible. Jeewon et al. [3] reported that *Pestalotiopsis* comprised two phylogenetic groups characterized by versicolorous or concolorous median conidial cells based on internal transcribed spacer (ITS) sequence data. Watanabe et al. (2012) [4] further differentiated these groups using ITS2 secondary structure analysis, classifying them as having umber-colored or pale-colored median cells. Subsequently, Maharachchikumbura et al. (2014) [5] identified three phylogenetically distinct groups with versicolorous, pale concolorous, and dark concolorous median cells using concatenated sequences of the ITS, β-tubulin, and translation elongation factor 1-alpha (*tef1*) regions. Therefore, they proposed elevating these three phylogenetic groups to generic rank as *Pestalotiopsis*, *Pseudopestalotiopsis*, and *Neopestalotiopsis*.

However, species that do not conform well to the defining characteristics of these genera have since been reported. For example, *Pestalotiopsis gibbosa* (Harkn.) Kyoko Watan., Nozawa & Callan [6] exhibits atypical morphological characteristics, making even genus-level identification difficult. These findings highlight the limitations of morphology-based identification and underscore the importance of molecular approaches for accurate genus delimitation.

Members of these genera have traditionally been isolated as endophytes from asymptomatic plant tissues [7,8,9], with the notable exception of tea gray leaf spot disease [10,11]. However, recent surveys have demonstrated that members of these genera can be serious pathogens of economically important crops, including loquat, strawberry, and grape [12,13,14]. Notably, *Neopestalotiopsis* species have been reported to exhibit stronger pathogenicity than *Pestalotiopsis* on loquat fruit [13]. These observations indicate that accurate identification at the genus level is essential for effective disease management.

Although *Pestalotiopsis* can be distinguished from *Neopestalotiopsis* and *Pseudopestalotiopsis* based on ITS length variation [15], reliable discrimination between *Neopestalotiopsis* and *Pseudopestalotiopsis* remains challenging and often requires additional sequence data. With the increasing availability of whole-genome data, comparative genomic analyses now enable the identification of genus-specific genes and nucleotide sequences. Therefore, this study aimed to develop genus-specific polymerase chain reaction (PCR) primers based on whole-genome data for the accurate identification of *Pestalotiopsis*, *Neopestalotiopsis*, and *Pseudopestalotiopsis*.

## 2. Materials and Methods

### 2.1. DNA Extraction for Genome Analysis

Genomic DNA was extracted from one strain of *Pestalotiopsis* sp. and eight strains of *Neopestalotiopsis* spp. using a modified cetyltrimethylammonium bromide (CTAB) method [16]. Each strain was cultured with shaking in yeast extract–glucose liquid medium (0.1% yeast extract and 0.1% glucose) for 7 d to obtain mycelial biomass. Approximately 0.4 g of dried mycelium was ground and suspended in 10 mL of 10% CTAB extraction buffer, followed by incubation at 60 °C for 40 min.

The lysate was divided into 1.5 mL microcentrifuge tubes (approximately 700 µL per tube), and an equal volume of chloroform:isoamyl alcohol (24:1) was added. The tubes were gently inverted for 10 min and centrifuged at 16,000× *g* for 10 min. The aqueous phase (approximately 600 µL) was transferred to a new tube and treated with 1 µL RNase A (100 mg/mL; Nippon Gene, Tokyo, Japan) at 37 °C for 1 h.

Following RNase treatment, an equal volume of chloroform:isoamyl alcohol (24:1) was added, and the samples were inverted for 10 min and centrifuged at 16,000× *g* for 10 min. The upper aqueous phase was transferred to a fresh tube. To remove polysaccharides and other contaminants, 0.1 volume of 3 M sodium acetate and 0.1 volume of 99.9% ethanol were added, followed by incubation on ice for 1 h and centrifugation at 16,000× *g* for 10 min. The supernatant was transferred to a new tube.

DNA was precipitated by adding 2.5 volumes of 99.9% ethanol; the mixture was gently mixed by inversion, incubated on ice for 30 min, and centrifuged at 14,000 rpm for 10 min. The resulting DNA pellet was washed with 1 mL of 80% ethanol and centrifuged at 16,000× *g* for 10 min. After removal of the ethanol and air-drying, the DNA was dissolved in 40 µL of AE buffer and stored for downstream analyses. Whole-genome sequencing was performed by Chemical Dojin Co., Ltd. (Kumamoto, Japan).

### 2.2. Genome-Scale Phylogenetic Analysis

To identify genus-specific genes in *Pestalotiopsis*, *Neopestalotiopsis*, and *Pseudopestalotiopsis*, the taxonomic placement of the strains used in this study was first confirmed through genome-scale phylogenetic analysis. Genomic data from 44 strains of *Pestalotiopsis*-related fungi preserved at Tamagawa University and the University of Guelph were used as the ingroup (Table S1). These strains belonged to the genera *Pestalotiopsis*, *Neopestalotiopsis*, and *Pseudopestalotiopsis* and had been identified in previous studies [13,17,18,19].

A *Seiridium* sp. strain (TAP1401) was used as the outgroup. Phylogenetic analysis was performed using a concatenation-based approach following the method reported by Nozawa et al. (2024) [20]. Gene prediction was conducted using Augustus v.3.3.3 [21] with the parameter “--species=fusarium.” Orthologous genes were identified by reciprocal BLASTP analysis using BLAST v.2.9.0+ with an E-value cutoff of 1 × 10^−5^. Amino acid sequences of each orthologous gene were aligned using Clustal Omega v.1.2.2 [22] with default settings.

Alignments longer than 1000 bp were retained after trimming gap-containing sites using trimAl v.1.2 [23]. A total of 1626 orthologous genes were concatenated, and phylogenetic trees were constructed using MEGA X with the neighbor-joining (NJ) method and 1000 bootstrap replicates [24].

### 2.3. Identification of Genus-Specific Genes and Primer Design

Genomic data from the 44 strains were used to identify candidate genus-specific genes for primer design. Gene prediction and orthology inference were conducted as described above. Candidate genes conserved within each genus but absent from the other two genera were selected and further verified by BLAST searches against the genomic data to confirm their genus specificity.

Primers were designed using Primer3Plus v.3.2.0 with default parameters for melting temperature (Tm), GC content, and primer length. Primer specificity was evaluated in silico using BLASTn searches against the NCBI nucleotide database.

### 2.4. Assessment of Primer Specificity Using Genomic DNA

To validate primer specificity, PCR assays were conducted using genomic DNA from 21 strains isolated from loquat, 15 strains from tea, one strain from Japanese andromeda, two strains from rubber, two strains from banana, and eight non-target strains belonging to *Colletotrichum, Fusarium*, *Seiridium*, and *Truncatella* (Table 1) [11,13,19,25,26]. DNA templates were extracted from mycelia grown on potato dextrose agar (PDA) for 7–10 d using the CTAB method described above [16]. PCR products were evaluated by agarose gel electrophoresis.

### 2.5. PCR Amplification and Sequencing of the ITS Region

To confirm successful DNA extraction, a set of ribosomal DNA regions, including the partial 18S rRNA gene, ITS1, the 5.8S rRNA gene, ITS2, and a partial region of the 28S rRNA gene (ITS), was amplified following the PCR conditions described by White et al. [27]. PCR was performed in a 10 µL reaction volume containing 7 µL of distilled water, 1 µL of 10× Ex *Taq* buffer with MgCl_2_, 0.8 µL of deoxynucleotide triphosphates (dNTPs; 10 mM each), 0.1 µL of each primer (50 µM), 0.05 µL of Ex Taq DNA polymerase (5 U µL^−1^; Takara, Tokyo, Japan), and 1.0 µL of genomic DNA template. PCR products were purified using ExoSAP-IT (GE Healthcare, Tokyo, Japan) and sequenced using a commercial Sanger sequencing service provided by FASMAC Co., Ltd. (Kanagawa, Japan).

### 2.6. Phylogenetic Analysis Based on ITS Sequences

To identify 41 strains at the genus level, phylogenetic analyses were conducted using nucleotide sequences of the ITS region. In addition to the strains obtained in this study, ITS sequences from reference strains comprising *Pestalotiopsis* (10 strains), *Neopestalotiopsis* (24 strains), and *Pseudopestalotiopsis* (7 strains) were included. A *Seiridium* sp. strain (TAP1401) was used as the outgroup.

Multiple sequence alignments were generated using ClustalW implemented in MEGA version 10 [28]. Phylogenetic trees were inferred using the NJ method with 1000 bootstrap replicates in MEGA 10.

### 2.7. Detection of Target Fungi from Plant Tissues

*Pestalotiopsis* sp. (TAP21H036) and *Neopestalotiopsis* sp. (TAP18N004), both of which are pathogenic to loquat, were cultured on PDA plates for 5 d. Mycelial plugs (5 mm in diameter) were excised from the margins of actively growing colonies and inoculated onto loquat leaves at six wounded sites per leaf. PDA plugs without mycelium were used as negative controls.

Leaf tissues (approximately 40 mg) were excised from the inoculated sites, and total DNA was extracted using the QIAamp DNA Mini Kit (Qiagen, Hilden, Germany) according to the manufacturer’s instructions. PCR amplification was carried out with an initial denaturation at 94 °C for 3 min, followed by 30–45 cycles of denaturation at 94 °C for 30 s, annealing at 65 °C for 30 s, and extension at 72 °C for 45 s, with a final extension at 72 °C for 7 min.

## 3. Results

### 3.1. Phylogenetic Relationships Among Pestalotiopsis, Neopestalotiopsis, and Pseudopestalotiopsis

**Figure 1:**
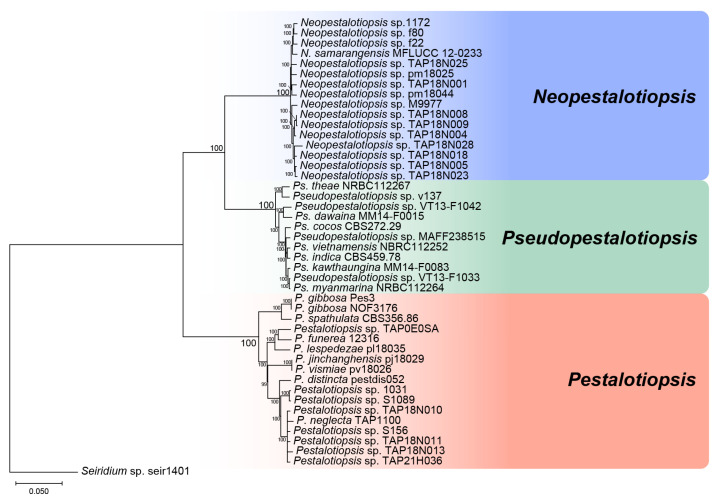
Phylogenetic tree based on the concatenated DNA sequence data of 1626 orthologues genes of the protein-coding region. Numbers on internal branches indicate 90% bootstrap (BS) values.

A total of 1626 orthologous genes were identified through reciprocal BLAST searches and used for genome-scale phylogenetic analysis. The concatenated nucleotide alignment of these genes comprised 2,481,365 bp. Phylogenetic inference based on the NJ method clearly resolved three well-supported clades corresponding to the genera *Pestalotiopsis*, *Neopestalotiopsis*, and *Pseudopestalotiopsis* (Figure 1).

The NJ tree recovered a monophyletic clade consisting of 17 strains of *Pestalotiopsis*, a second clade comprising 16 strains of *Neopestalotiopsis*, and a third clade containing 11 strains of *Pseudopestalotiopsis*. All three clades were supported by 100% bootstrap values, indicating robust phylogenetic separation among the three genera.

### 3.2. Specificity of Gene Regions for Primer Design and Primer Profiles

The primers used in this study were not designed from conventional phylogenetic barcode regions such as ITS, tef1-α, or β-tubulin. Instead, they were developed from novel genus-specific coding loci identified through genome-based orthology analysis.

#### 3.2.1. Neopestalotiopsis

**Figure 2:**
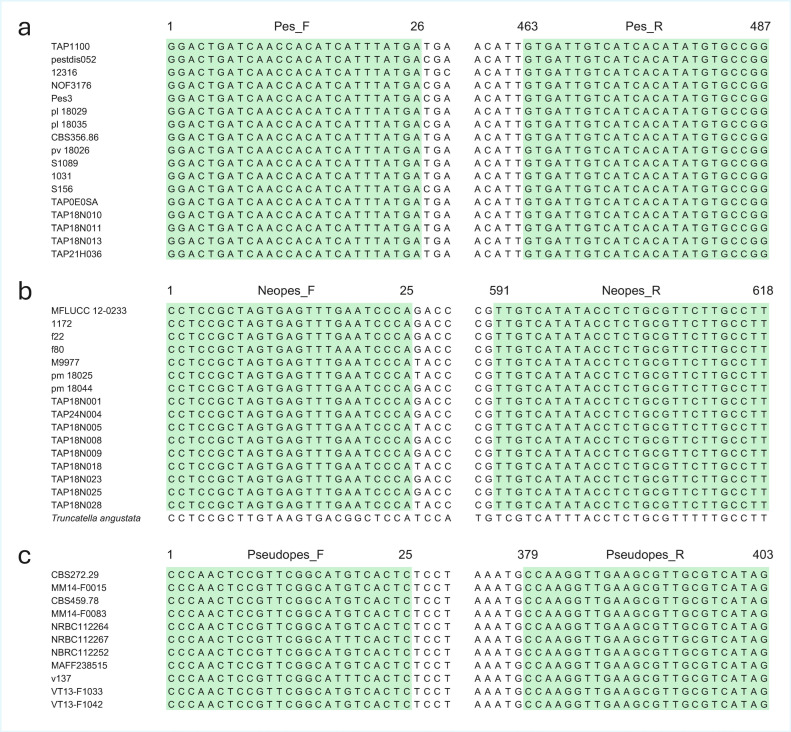
Alignment of primer binding sites on target sequences of *Pestalotiopsis* strains (**a**), *Neopestalotiopsis* strains (**b**), and *Pseudopestalotiopsis* strains (**c**). Highlighted sites are primer sites.

For the genus *Neopestalotiopsis*, predicted gene 4658 from *Neopestalotiopsis* sp. TAP18N004 was selected as the optimal candidate for primer design. A BLAST search using the target gene sequence as a query showed that the only hit outside the target genus was *Truncatella angustata* (sequence ID: XM_046107504; Figure 2b). However, the *T. angustata* sequence did not contain primer binding sites within the region targeted by the primers.

Based on gene 4658, the primer pair Neopes_F (5′-CCTCCGCTAGTGAGTTTGAATCCCA-3′) and Neopes_R (5′-AAGGCAAGAACGCAGAGGTATATGACAA-3′) was designed to amplify a 618 bp fragment. The GC contents and Tm of Neopes_F and Neopes_R were 52.0% and 42.8%, and 71.5 °C and 70.4 °C, respectively. The primer binding sites were completely conserved among all 16 *Neopestalotiopsis* strains analyzed (Figure 2b).

#### 3.2.2. Pestalotiopsis

For the genus *Pestalotiopsis*, predicted gene 13712 from *Pestalotiopsis* sp. TAP21H036 was selected for primer design. A BLAST search of the target sequence against the NCBI nucleotide database returned no significant hits outside the genus (Figure 2a).

The primer pair Pes_F (5′-GGACTGATCAACCACATCATTTATGA-3′) and Pes_R (5′-CCGGCACATATGTGATGACAATCAC-3′) was designed to amplify a 487 bp fragment. The GC contents and Tm of Pes_F and Pes_R were 38.4% and 48.0%, and 66.5 °C and 70.8 °C, respectively. The primer binding sites were completely conserved across all 17 *Pestalotiopsis* strains examined (Figure 2a).

#### 3.2.3. Pseudopestalotiopsis

For the genus *Pseudopestalotiopsis*, predicted gene 9466 from *Pseudopestalotiopsis theae* (NRBC 112267) was selected for primer design. BLAST analysis indicated that the only hit was *Pseudopestalotiopsis fici* (Figure 2c).

Based on this gene, the primer pair Pseudopes_F (5′-CCCAACTCCGTTCGGCATK TCACTC-3′) and Pseudopes_R (5′-CTATGACGCAACGCTTCAACCTTGG-3′) was designed to amplify a 403 bp fragment. The GC contents and Tm of Pseudopes_F and Pseudopes_R were 56.0% and 52.0%, and 75.3 °C and 72.6 °C, respectively. The primer binding sites were conserved across 11 *Pseudopestalotiopsis* strains, except for a single nucleotide variation observed in one strain (Figure 2c).

#### 3.2.4. Unified PCR Conditions

To allow amplification using all primer pairs under a single PCR program, a unified thermal cycling protocol was applied: an initial denaturation at 94 °C for 3 min, followed by 30 cycles of denaturation at 94 °C for 30 s, annealing at 55 °C for 20 s, and extension at 72 °C for 45 s, with a final extension at 72 °C for 7 min.

### 3.3. Assessment of Primer Performance

To evaluate the specificity and accuracy of the developed primers, strains belonging to three *Pestalotiopsis*-related genera obtained from loquat, tea, Japanese andromeda, rubber, and banana were tested. Successful DNA extraction from all strains was confirmed by amplification of the ITS region (Table 1; Figures S1 and S2).

The primer pair Neopes_F and Neopes_R, designed for the specific detection of the genus *Neopestalotiopsis*, successfully amplified PCR products of approximately 600 bp in all 24 *Neopestalotiopsis* strains tested, which were isolated from loquat, Japanese andromeda, and tea (Table 1; Figure S2).

**Figure 3:**
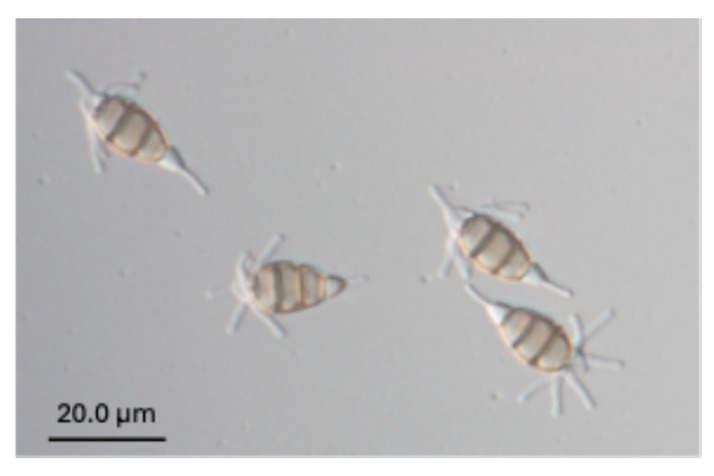
*Pestalotiopsis* (TAP21H062) exhibited atypical morphological characteristics. The apical appendages of the conidia originated from the basal region of the apical cell.

Similarly, the primer pair Pes_F and Pes_R, targeting the genus *Pestalotiopsis*, produced PCR amplicons of approximately 500 bp in 9 out of 10 *Pestalotiopsis* strains derived from loquat. (Table 1; Figure S2). The single strain (TAP21H062, Figure 3) that was not detected was phylogenetically distinct and exhibited atypical morphological characteristics, in which the apical appendages of the conidia originated from the basal region of the apical cell [19].

For the genus *Pseudopestalotiopsis*, the primer pair Pseudopes_F and Pseudopes_R generated PCR products of approximately 400 bp in all seven tested strains isolated from loquat, rubber, and banana (Table 1; Figure S2).

**Figure 4:**
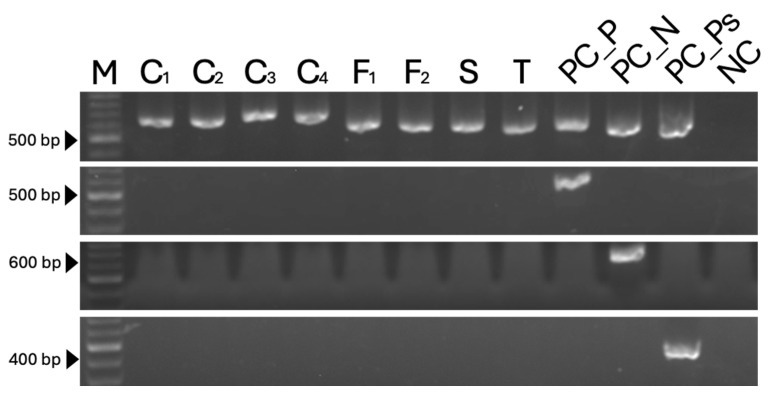
PCR analysis of non-target taxa using each genus-specific primer set. The panels show the results of PCR analysis using the primer sets ITS5/4, PesF_Pes_R, Neopes_F/Neopes_R, and Pseudopes_F/Pseudopes_R from top to bottom. M: marker sizes using the Excel-Band™ 100 bp DNA ladder (SMOBIO, Hsinchu, Taiwan); C_1–4_: *Colletotrichum* strains (TAP19T001, TAP19T002, TAP19T021, and TAP20T033); F_1–2_: *Fusarium* strains (TAP21H006 and TAP21H007); S: *Seiridium* strain (TAP21H020); T: *Truncatella* (H462). PC_P: positive control from *Pestalotiopsis* (TAP21H036); PC_N: positive control from *Neopestalotiopsis* (TAP18N004); PC_Ps: positive control from *Pseudopestalotiopsis* (TAP21H021); NC: negative control with double-distilled water (ddH_2_O).

None of the primer sets amplified DNA from non-target taxa, including *Colletotrichum*, *Fusarium*, *Seiridium*, and *Truncatella*, nor from the other two *Pestalotiopsis*-related genera, demonstrating high genus specificity. No amplification was observed in negative controls containing sterile water as the PCR template (Figure 4).

### 3.4. Detection from Infected Leaves

**Figure 5:**
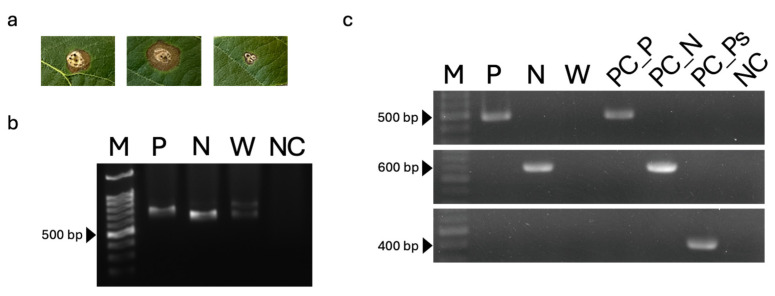
PCR-based detection of *Pestalotiopsis* and *Neopestalotiopsis* from inoculated plant tissues. (**a**) Loquat leaves inoculated with *Pestalotiopsis* sp. (TAP21H036; **left**), *Neopestalotiopsis* sp. (TAP18N004; **middle**), and sterile water as a negative control (**right**). (**b**) PCR amplification of the ITS region using template DNA extracted from leaves inoculated with *Pestalotiopsis* sp. (lane P), *Neopestalotiopsis* sp. (lane N), and sterile water (lane W). (**c**) PCR analysis using genus-specific primers and template DNA extracted from leaves inoculated with *Pestalotiopsis* sp. (lane P), *Neopestalotiopsis* sp. (lane N), and sterile water (lane W). Lanes PC_P, PC_N, and PC_Ps represent positive controls using genomic DNA extracted from mycelia of *Pestalotiopsis* sp. (TAP21H036), *Neopestalotiopsis* sp. (TAP18N004), and *Pseudopestalotiopsis* sp. (TAP21H021), respectively. The **upper**, **middle**, and **lower** panels in (**c**) correspond to PCR results obtained using primers specific for the genera *Pestalotiopsis*, *Neopestalotiopsis*, and *Pseudopestalotiopsis*, respectively. Sterile distilled water (ddH_2_O) was used as a negative control (lane NC). The first lane in each panel (M) indicates the molecular size marker, ExcelBand™ 100 bp DNA ladder (SMOBIO).

PCR amplification of the ITS region was first performed using DNA extracted from symptomatic loquat leaf tissues as the template (Figure 5a). Amplification of an approximately 500 bp fragment confirmed successful DNA extraction from the infected leaf tissues (primer diagram shown in Figure 5b).

Subsequently, genus-specific PCR assays were conducted using the developed primer sets. Amplicons of the expected sizes were detected exclusively with the corresponding genus-specific primers: a 500 bp fragment was amplified only from leaf tissues inoculated with *Pestalotiopsis* using the *Pestalotiopsis*-specific primer pair Pes_F and Pes_R, whereas a 600 bp fragment was amplified only from leaf tissues inoculated with *Neopestalotiopsis* using the *Neopestalotiopsis*-specific primer pair Neopes_F and Neopes_R (Figure 5c).

Using the *Pestalotiopsis*-specific primers Pes_F and Pes_R, PCR products were obtained exclusively from diseased leaf tissues and fungal DNA extracted from leaves inoculated with *Pestalotiopsis* sp. Likewise, the *Neopestalotiopsis*-specific primers Neopes_F and Neopes_R yielded PCR products only from diseased tissues and fungal DNA derived from *Neopestalotiopsis*-inoculated leaves.

No amplification was observed from tissues inoculated with sterile water, from negative controls using sterile water as the PCR template, or when *Pseudopestalotiopsis*-specific primers were applied. These results demonstrate that the developed genus-specific primers enable reliable and direct detection of *Pestalotiopsis* and *Neopestalotiopsis* pathogens from infected loquat leaf tissues.

## 4. Discussion

Although the genera *Pestalotiopsis*, *Neopestalotiopsis*, and *Pseudopestalotiopsis* were established in 2014 based on differences in the coloration of the three median conidial cells, species that do not conform to these diagnostic characteristics have subsequently been reported [5,6,29,30]. Moreover, reliable discrimination between *Neopestalotiopsis* and *Pseudopestalotiopsis* remains difficult without DNA sequence data. In practice, these genera are frequently co-isolated from similar lesion types, and morphological overlap often prevents accurate genus-level identification based solely on phenotypic traits.

Fungi belonging to these genera have been increasingly reported as pathogens of economically important crops, including tea, loquat, strawberry, guava, blueberry, and rubber tree [12,13,14,31,32]. In particular, rubber trees represent a major cash crop in tropical regions, and disease outbreaks associated with these genera have raised significant economic concerns. In several agricultural systems, species from two or more of these genera may be isolated from similar disease symptoms on the same host, making accurate diagnosis particularly challenging. Rapid and reliable genus-level discrimination is therefore essential not only for taxonomic clarification but also for effective disease management, source tracking of infection, and large-scale processing of field samples during disease outbreaks.

In the present study, we developed genus-specific PCR primers and demonstrated that the three genera could be accurately detected and distinguished at the genus level based solely on the presence or absence of PCR amplicons visualized by gel electrophoresis.

This approach enables clear genus-level identification even in cases where morphological traits overlap and where the ITS amplicon length obtained using universal primers does not allow discrimination between *Neopestalotiopsis* and *Pseudopestalotiopsis*. Amplification of the ITS region from DNA extracted directly from lesion tissues does not allow reliable discrimination among these genera based solely on amplicon size; in most cases, sequencing is required to achieve accurate genus-level identification and to exclude other co-occurring fungal taxa, increasing both time and cost. The significance of this method lies in its ability to provide a practical and reliable framework for genus-level identification, which is essential for both taxonomic studies and applied plant pathology. Importantly, no amplification was observed in other common phytopathogenic genera such as *Colletotrichum* and *Fusarium* (Table 1), supporting the diagnostic specificity of the developed primers.

In contrast to pathogens with strict host specificity, such as *Fusarium oxysporum* f. sp. *cubense* tropical race 4, for which race-specific or forma specialis–specific PCR primers have been developed [20,33,34,35], multiple species within *Pestalotiopsis*, *Neopestalotiopsis*, and *Pseudopestalotiopsis* can act as pathogens on a single host plant. Furthermore, inconsistencies between morphological characteristics and molecular phylogenetic placement [11,13], as well as poorly resolved phylogenetic relationships [36], have resulted in strains that remain taxonomically ambiguous. Under these circumstances, genus-level identification represents a critical and realistic resolution for plant disease management and epidemiological studies. Given their taxonomic complexity and occurrence on economically important crops, a sequencing-free and scalable identification framework is particularly valuable.

In this study, genus-level identification was accurate for all *Pestalotiopsis* strains examined, except for a single strain. This undetected strain did not cluster within the main *Pestalotiopsis* clade in the phylogenetic tree and exhibited distinct conidial morphology, characterized by the presence of multiple appendages between the apical and the second cells of the conidium. Notably, this species has not been reported as a plant pathogen, and the strains examined in this study did not exhibit pathogenicity to loquat, the host from which they were isolated. These findings further support the taxonomic validity and practical utility of the genus-specific primer system developed in this study.

Recent advances in diagnostic technologies, such as loop-mediated isothermal amplification, have enabled rapid pathogen detection [37]. However, the primary objective of the present study was not rapid diagnosis per se, but the accurate taxonomic assignment of a large and diverse set of strains into the three recognized genera. To achieve this goal, it was essential to classify numerous strains simultaneously at the genus level using a unified analytical framework. While the PCR-based method developed here successfully fulfills this objective, further improvement of rapid and simple DNA extraction methods from diseased tissues of woody plants will be required to facilitate future field-level applications. Adaptation of the identified genus-specific loci to qPCR platforms represents a logical next step to enhance diagnostic sensitivity and expand practical applicability. Further validation using naturally infected plant tissues will require accumulation of well-characterized field samples and careful evaluation under complex infection conditions.

## 5. Conclusions

In this study, we developed and validated genus-specific PCR primers that enable reliable identification of *Pestalotiopsis*, *Neopestalotiopsis*, and *Pseudopestalotiopsis* at the genus level. By integrating genome-scale comparative analyses with primer design, the developed primer sets successfully discriminated the three morphologically similar genera based on simple PCR amplification and gel electrophoresis. The primers demonstrated high specificity across a broad range of strains obtained from multiple host plants and allowed direct detection of target fungi from infected plant tissues without the need for fungal isolation. Given the frequent overlap of morphological traits, the involvement of multiple species on a single host, and unresolved phylogenetic relationships within these genera, genus-level identification represents a practical and effective approach for plant disease diagnosis and epidemiological studies. The PCR-based method presented here provides a robust taxonomic framework and a useful diagnostic tool for both fundamental mycological research and applied plant disease management.

## Figures and Tables

**Table 1:** List of fungal strains used to assess developed PCR primers and PCR results.

Genus	Source	Strain No.	Primer Sets		
			Pes_F	Neopes_F	Pseudopes_F
			Pes_R	Neopes_R	Pseudopes_R
*Pestalotiopsis*	Loquat	TAP18N013	+	−	−
		TAP18N014	+	−	−
		TAP18P010	+	−	−
		TAP20N03	+	−	−
		TAP20N06	+	−	−
		TAP21H029	+	−	−
		TAP21H036	+	−	−
		TAP21H038	+	−	−
		TAP21H041	+	−	−
		TAP21H062	−	−	−
*Neopestalotiopsis*	Tea	SPPTPkawa2	−	+	−
		SPPTPko1	−	+	−
		SPPTPko2	−	+	−
		SPPTPko3	−	+	−
		SPPTPko4	−	+	−
		SPPTPko5	−	+	−
		SPPTPko6	−	+	−
		SPPTPko7	−	+	−
		SPPTPmaki1	−	+	−
		SPPTPmaki2	−	+	−
		SPPTPmaki4	−	+	−
		SPPTPmaki5	−	+	−
		SPPTPmaki6	−	+	−
		SPPTPshizu1	−	+	−
		SPPTPshizu2	−	+	−
	Japanese andromeda	TAP16NN	−	+	−
	Loquat	TAP18N001	−	+	−
		TAP18N003	−	+	−
		TAP18N004	−	+	−
		TAP18N005	−	+	−
		TAP18N018	−	+	−
		TAP18N022	−	+	−
		TAP18N024	−	+	−
		TAP21H024	−	+	−
*Pseudopestalotiopsis*	Rubber	PH12-1278	−	−	+
		PH12-1279	−	−	+
	Loquat	TAP21H021	−	−	+
		TAP21H022	−	−	+
		TAP21H023	−	−	+
	Banana	TAP24N051	−	−	+
		TAP24N119	−	−	+
*Colletotrichum*	Loquat	TAP19T001	−	−	−
		TAP19T002	−	−	−
		TAP19T021	−	−	−
		TAP20T033	−	−	−
*Fusarium*	Loquat	TAP21H006	−	−	−
		TAP21H007	−	−	−
*Seiridium*	Loquat	TAP21H020	−	−	−
*Truncatella*	–	H462	−	−	−

+, indicating that the strain was detected with the primer sets. −, indicating that the strain was not detected with the primer sets. PCR, polymerase chain reaction.

## Data Availability

The original contributions presented in this study are included in the article/Supplementary Material. Further inquiries can be directed to the corresponding author.

## Supplementary Materials

The following supporting information can be downloaded at: https://www.mdpi.com/article/10.3390/jof12030198/s1, Figure S1. The phylogenetic tree was constructed using nucleotide sequences of the internal transcribed spacer (ITS) region with the neighbor-joining (NJ) method implemented in MEGA version 10. Bootstrap values (>50%) based on 1000 replicates are shown at the internal branches. An asterisk (*) following a strain number indicates an ex-type or type strain, with the corresponding accession number shown in parentheses. A hyphen (–) indicates that the sequence was newly generated in this study. Symbols at the end of strain names indicate the host plant from which each isolate was obtained: ● loquat, ▲ tea, ■ Japanese andromeda, ○ rubber, and □ banana. Figure S2. PCR analysis of strains isolated from loquat, tea, Japanese andromeda, rubber and banana using each primer set. PCR analysis of strains of loquat, tea, Japanese andromeda, rubber and banana using the primer set Pes_F/R, Neopes_F/R and Pseudopes_F/R. Strain names were shown on each lane. Each panel’s first lane (M) show marker size using the ExcelBand™ 100 bp DNA ladder (SMOBIO). Four panels are the result of the amplification of ITS, gene12709 of *Pestalotiopsis*, gene4658 of *Neopestalotiopsis* and gene9466 of *Pseudopestalotiopsis* regions, respectively. Strains with red character indicate that the target region was amplified by the primer set Pes_F/R. Strains with red circle indicate that isolates were identified as *Pestalotiopsis* sp. Strains with blue character indicate that the target region was amplified by the primer set Neopes_F/R. Strains with blue triangle indicate that isolates were identified as *Neopestalotiopsis* sp. Strains with green character indicate that the target region was amplified by the primer set Pseudopes_F/R. Strains with green square indicate that isolates were identified as *Pseudopestalotiopsis* sp. Lane PC_P is positive control from TAP21H036 (*Pestalotiopsis* sp.). Lane PC_N is positive control from TAP18N004 (*Neopestalotiopsis* sp.). Lane PC_Ps is positive control from TAP21H021 (*Pseudopestalotiopsis* sp.). Lane NC is negative control with ddH_2_O. Table S1: The list of used genome data.

## Abbreviations

The following abbreviations are used in this manuscript:

ITS	Internal transcribed spacer
PCR	Polymerase chain reaction
CTAB	Cetyltrimethylammonium bromide
PDA	Potato dextrose agar
dNTP	Deoxynucleotide triphosphates
NJ	Neighbor-joining
Tm	Melting temperature
