# Supplementary material for: Genome-Based Development of Genus-Specific PCR Primers for Pestalotiopsis, Neopestalotiopsis, and Pseudopestalotiopsis
Source: J Fungi (Basel). 2026 Mar 10;12(3):198. doi: 10.3390/jof12030198 (PMC13028432; doi:10.3390/jof12030198)
Supplement: Supplementary file 1 [file jof-12-00198-s001.zip › Figure S2.pptx]

## Slide 1
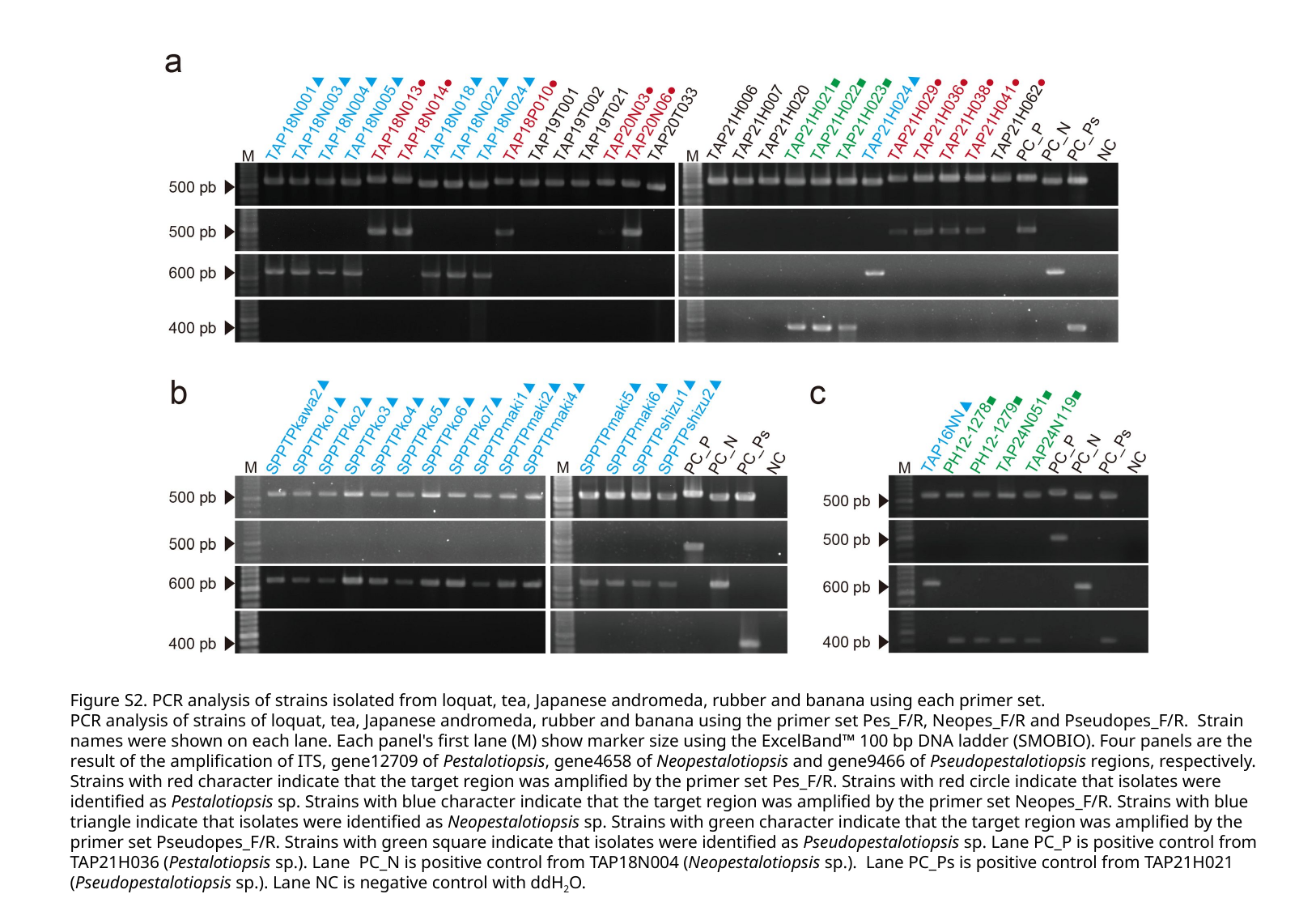

Figure S2. PCR analysis of strains isolated from loquat, tea, Japanese andromeda, rubber and banana using each primer set.
PCR analysis of strains of loquat, tea, Japanese andromeda, rubber and banana using the primer set Pes_F/R, Neopes_F/R and Pseudopes_F/R. Strain names were shown on each lane. Each panel's first lane (M) show marker size using the ExcelBand™ 100 bp DNA ladder (SMOBIO). Four panels are the result of the amplification of ITS, gene12709 of Pestalotiopsis, gene4658 of Neopestalotiopsis and gene9466 of Pseudopestalotiopsis regions, respectively. Strains with red character indicate that the target region was amplified by the primer set Pes_F/R. Strains with red circle indicate that isolates were identified as Pestalotiopsis sp. Strains with blue character indicate that the target region was amplified by the primer set Neopes_F/R. Strains with blue triangle indicate that isolates were identified as Neopestalotiopsis sp. Strains with green character indicate that the target region was amplified by the primer set Pseudopes_F/R. Strains with green square indicate that isolates were identified as Pseudopestalotiopsis sp. Lane PC_P is positive control from TAP21H036 (Pestalotiopsis sp.). Lane PC_N is positive control from TAP18N004 (Neopestalotiopsis sp.). Lane PC_Ps is positive control from TAP21H021 (Pseudopestalotiopsis sp.). Lane NC is negative control with ddH2O.
