# Supplementary figures and images for: Genome-Based Development of Genus-Specific PCR Primers for Pestalotiopsis, Neopestalotiopsis, and Pseudopestalotiopsis
Source: J Fungi (Basel). 2026 Mar 10;12(3):198. doi: 10.3390/jof12030198 (PMC13028432; doi:10.3390/jof12030198)

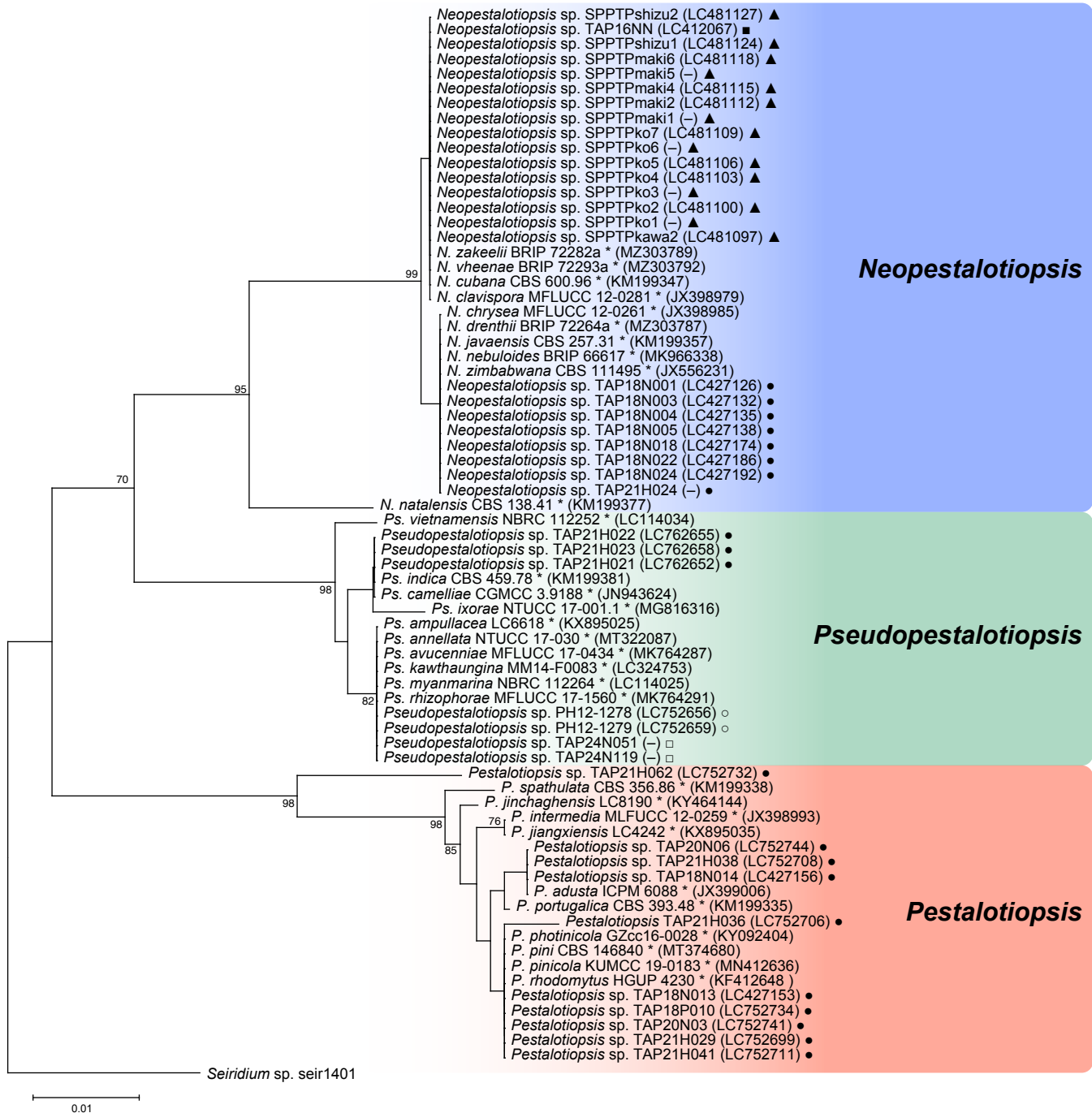

Supplement: Supplementary file 1 [file jof-12-00198-s001.zip › Figure S1.pdf]
